# Polyphenols derived from *Sorghum bicolor* grains exhibit the ability to alleviate amyloid beta in Alzheimer’s Disease

**DOI:** 10.1002/alz.088593

**Published:** 2025-01-09

**Authors:** Rasheed A. Abdulraheem, Prashant Bharadwaj, Zhaoyu Li, Ranil Coorey, Stuart Johnson, Ralph N Martins, Warnakulasuriya Mary Ann Dipika Binosha Fernando

**Affiliations:** ^1^ Edith Cowan University, Perth, Western Australia Australia; ^2^ Australian Alzheimer’s Research Foundation, Perth, Western Australia Australia; ^3^ The University of Queensland Brain Institute, Brisbane, QLD Australia; ^4^ Curtin University, Perth, Western Australia Australia; ^5^ IngredientsbyDesign Pty, Perth, Western Australia Australia; ^6^ Macquarie University, Sydney, NSW Australia; ^7^ Australian Alzheimer's Research Foundation, Perth, Western Australia Australia

## Abstract

**Background:**

Accumulation of amyloid beta 42 (Aβ42) senile plaques is the most critical event leading to Alzheimer’s disease (AD). Currently approved drugs for AD have not been able to effectively modify the disease. This has caused increasing research interests in health beneficial nutritious plant foods as viable alternative therapy to prevent or manage AD. *Sorghum bicolor* presents strong antioxidants, anticancer, anti‐inflammatory and antidiabetic properties. However, there is no published data on how this widely available and cost‐effective food could benefit people living with AD.

**Method:**

Polyphenols were extracted from three Sorghum varieties with black, reddish‐brown, and red pericarp. The individual polyphenols compounds were identified using an Agilent 1290 infinity 2 UHPLC system coupled to an Agilent 6475 LC‐QQQ LC‐MS/MS. The polyphenol extracts were screened using *in‐silico*, MC65 cell and *Caenorhabditis elegans* AD models. The activities of the extracts against Aβ42 aggregation were analyzed using molecular docking, fluorescence, toxicity, and healthspan assays.

**Result:**

Through the application of molecular docking, polyphenols extracted from Sorghum bicolor grains were identified for their potential to inhibit Aβ42 aggregation. Positive interactions were observed between the polyphenols and Aβ42. Cell studies indicated increased viability of MC65 cells, possibly attributed to the inhibition of Aβ42 aggregation. In the Alzheimer’s disease model of Caenorhabditis elegans, enhanced body bending, and pharyngeal pumping were observed compared to controls, suggesting a reduction in pathology. The order of activities was found to be red pericarp < reddish‐brown pericarp < black pericarp sorghum.

**Conclusion:**

The polyphenol extracts from black pericarp *Sorghum bicolor* grain could be a candidate ingredient in the development of complementary dietary supplement for AD therapy. Future investigation include lifespan, and fluorescence imaging to unravel the mechanisms underlying the observed health benefits prior to study in AD animal models.

